# The double-edged role of SPAG6 in male reproductive toxicity: pro-invasive in leukemia, protective in germ cells

**DOI:** 10.3389/fmed.2026.1855817

**Published:** 2026-08-27

**Authors:** Jing Luo, Jiaxiu Yin, Junli Mou, Kexin Lin, Lin Liu, Yao Zhang

**Affiliations:** 1 Department of Hematology, The First Affiliated Hospital of Chongqing Medical University, Chongqing, China; 2 Department of Hematology, The Affiliated Hospital of North Sichuan Medical College, Nanchong, Sichuan Province, China

**Keywords:** acute myeloid leukemia, chemotherapy, oxidative stress, reproductive protection, SPAG6 gene

## Abstract

**Background:**

Male testis is highly susceptible to irreversible damage from acute myeloid leukemia (AML) infiltration and subsequent chemotherapy. As a gene critical for sperm function, the role of SPAG6 in AML testicular infiltration and chemotherapy-induced injury remains poorly elucidated.

**Methods:**

SPAG6 expression was knocked down in C1498 leukemia cell line, and its impact on testicular infiltration was evaluated *in vivo*. RNA sequencing was performed to identify altered pathways. In parallel, SPAG6 was silenced in murine GC-1 spg spermatogonial cells, followed by assessment of reactive oxygen species (ROS) levels and cell proliferation.

**Results:**

Both AML infiltration and subsequent chemotherapy exerted profound damage on the testes. SPAG6 was highly expressed in normal mouse testes and was upregulated upon AML infiltration and chemotherapy. Knockdown of SPAG6 in leukemia cells was associated with suppressed proliferation and induced apoptosis *in vitro*, and reduced testicular infiltration *in vivo*; chemotherapy further enhanced these effects. RNA sequencing and wound healing assay further indicated that SPAG6 depletion may impair cell adhesion and migration. Conversely, silencing SPAG6 in spermatogonial cells was linked to suppressed proliferation, elevated mitochondrial membrane potential and ROS levels, and these effects were further exacerbated under chemotherapeutic challenge.

**Conclusion:**

SPAG6 may exert cell-type-specific, opposing roles in AML-infiltrated testes: SPAG6 depletion is associated with suppressed leukemic cell infiltration, potentially through modulation of cell adhesion, while its deficiency correlates with increased chemotherapy-induced injury to spermatogonial cells.

## Introduction

Acute myeloid leukemia (AML), the most common type of acute leukemia in adults, characterized by accumulation of immature myeloid cells and hematopoietic suppression in the bone marrow (1). Beyond the bone marrow, leukemic cells frequently infiltrate multiple organs, including the liver, spleen, lymph nodes, brain, and reproductive system (2–4). While chemotherapy remains the cornerstone of AML treatment and has significantly improved patient survival, both leukemic infiltration and the chemotherapeutic agents themselves can severely damage the reproductive system (5–7). Currently, most research emphasizes extending patient survival, leaving the preservation of quality of life and reproductive health largely overlooked. The male reproductive organs, testes, are highly proliferative and particularly sensitive to chemotherapeutic agents, which can induce testicular impairment through the disruption of the blood–testis barrier (BTB), reactive oxygen species (ROS)-driven mitochondrial dysfunction, and the irreversible depletion of spermatogonial stem cells (5, 8). Despite the availability of protective strategies such as sperm cryopreservation for sexually mature males, the pathological mechanisms underlying testicular damage in leukemia remain poorly understood, and effective interventions are still lacking.

Sperm-associated antigen 6 (SPAG6), located on human chromosome 10p12, encodes a microtubule-associated protein and belongs to the cancer-testis antigen (CTA) family (9). SPAG6 is an axonemal central pair protein that regulates flagellar and ciliary motility. Under physiological conditions, SPAG6 is highly and exclusively expressed in testicular tissue, where it contributes to sperm motility, ciliary function, and maintenance of testicular architecture (10, 11). However, as a typical cancer-testis antigen, SPAG6 is also implicated in the initiation and progression of various human malignancies, including breast cancer, osteosarcoma, myelodysplastic syndrome, and AML (9, 12, 13). In hematological malignancies, Steinbach et al. (14) reported that SPAG6 expression is markedly upregulated in AML patients but returns to normal levels upon achieving sustained complete remission. Our previous studies further demonstrated that high SPAG6 expression correlates with poor prognosis in leukemia, mechanistically revealing that SPAG6 activates the PI3K/AKT and ERK signaling pathways to promote AML cell proliferation and disease progression (15–17). Given the critical roles of SPAG6 in both spermatogenesis and leukemia progression, it remains elusive whether SPAG6 is involved in testicular injury induced by leukemic infiltration and chemotherapy.

This study aims to elucidate the role of SPAG6 in leukemic infiltration and chemotherapy-induced testicular impairment. We found that SPAG6 knockdown in leukemia cells was associated with attenuated testicular infiltration, potentially through modulation of cell adhesion pathways. Conversely, SPAG6 silencing in spermatogonial cells correlated with increased ROS accumulation and suppressed cell proliferation. Collectively, these findings suggest that SPAG6 may exert cell-type-specific, opposing roles in AML-infiltrated testes: it appears to contribute to the progression of leukemic cells while concurrently serving a protective function against chemotherapy-induced injury to the testis.

## Methods

### Mouse model

A total of 2 × 10^5^ wildtype C1498 cell lines were injected via the tail vein into C57BL/6J male mice to establish the leukemia model. The mice were then randomly assigned to two groups: one receiving concurrent cytarabine treatment, while the other receiving subcutaneous PBS. Age-matched healthy littermates served as the control. On day 21 post-injection, mice were euthanized, and testicular tissues were harvested for subsequent histological evaluation via hematoxylin and eosin (H&E) staining and immunohistochemical (IHC) analysis.

### Migration assays

GC-1 spg cells were seeded into 6-well plates at a density of 1 × 106 cells per well. After initial attachment, the cells were co-cultured with either DMEM, C1498 cells (0.5 × 10^6^ cells per well), or Ara-C-treated C1498 cells for an additional 24 h. Subsequently, the GC-1 spg cells were trypsinized and transferred to the upper chamber of a 24-well Transwell insert in serum-free medium. The lower chamber was supplied with 20% FBS-supplemented medium to induce chemotaxis. After 24 h, migrated cells on the lower surface were fixed, stained with 0.1% crystal violet, and quantified via light microscopy.

### RNA sequencing

Total RNA was extracted from 1 × 10^6^ cells using 1 mL of TRIzol reagent, and samples were submitted to Novogene (Beijing, China) for library construction and high-throughput sequencing. RNA integrity was verified using the RNA Nano 6000 Assay Kit on the Agilent Bioanalyzer 2100 system. Raw data in FASTQ format were processed via custom Perl scripts to generate clean reads by removing adapter-containing sequences, poly-N sequences, and low-quality reads. All subsequent analyses were performed using these high-quality clean data. Gene expression levels were quantified using featureCounts to obtain read counts mapped to each gene, which were then normalized to Fragments Per Kilobase of transcript per Million mapped reads (FPKM). Differential expression analysis was performed using the DESeq2 R package (v1.20.0). Genes exhibiting an adjusted *p*-value < 0.05 and a |log2(Fold Change)| > 1 were identified as differentially expressed genes (DEGs).

### Cell counting kit 8 (CCK-8) assay

Cell proliferation was assessed using the Cell Counting Kit-8 (MedChemExpress) according to the manufacturer's protocol. Briefly, cells from each group were seeded into 96-well plates at a density of 6 × 10^3^ cells per well in 100 μL of culture medium. At 0, 24, 48, and 72 h post-seeding, 10 μL of CCK-8 reagent was added to each well and incubated for additional 3 h at 37 °C. The absorbance at 450 nm was subsequently measured using a microplate reader.

### Cell apoptosis and cell cycle analysis

For apoptosis evaluation, cells were harvested, washed, incubated with DAPI-PB and propidium iodide (PI) using the DAPI-PB/PI Apoptosis Detection Kit (BD Biosciences, USA). For cell cycle assessment, cells were pulse-labeled with bromodeoxyuridine (BrdU), fixed, permeabilized, and stained with anti-BrdU antibody and 7-aminoactinomycin D (7-AAD) using the BD Pharmingen™ BrdU Flow Kit (BD Biosciences, USA). Following the respective staining procedures, samples were analyzed via flow cytometry. All data were subsequently processed and quantified using FlowJo software.

### Lentiviral transduction and stable cell line generation

Lentiviral vectors expressing SPAG6-shRNA were obtained from GenePharma (Shanghai, China), and transduction was performed following the manufacturer's protocols. At 48 h post-transduction, cells were subjected to 2 μg/mL puromycin selection to generate stable cell lines. Then the selection medium was replenished every 2–3 days for a duration of 5–10 days until all non-transduced control cells were eliminated and surviving cells exhibited stable growth. Subsequently, stable cell pools were maintained in medium containing a maintenance dose of puromycin (0.5 μg/mL) for further experiments.

### Intracellular ROS assay

Intracellular reactive oxygen species (ROS) production was evaluated using a commercial ROS Assay Kit (Beyotime, Shanghai, China) according to the manufacturer's instructions. Briefly, GC-1 spg cells were incubated with 10 μM DCFH-DA fluorescent probe at 37 °C for 30 min in the dark. Following thorough washing to eliminate background fluorescence, the cells were promptly analyzed via flow cytometry. All samples were acquired under identical instrument settings, and the ROS levels were quantified based on the mean fluorescence intensity using FlowJo software.

### Mitochondrial membrane potential assessment

Mitochondrial membrane potential (MITO) was evaluated using a MitoTracker staining kit (Beyotime, Shanghai, China) according to the manufacturer's protocol. Briefly, GC-1 spg cells were harvested, washed with PBS, and incubated with MitoTracker working solution at 37 °C for 10 min in the dark. Following incubation, cells were washed twice to remove unbound dye and immediately analyzed by flow cytometry. Fluorescence signals were captured in the APC channel and MITO was quantified based on the mean fluorescence intensity using FlowJo software.

### Wound healing assay

GC-1 spg cells were seeded in 6-well plates at a density of 1 × 10^6^ cells per well and cultured until reaching confluence. A linear scratch was created across the cell monolayer using a sterile 200-μL pipette tip, and detached cells were removed by washing with PBS. Transwell inserts (0.4-μm pore size) were subsequently placed into the wells. 5 × 10^5^ control or SPAG6-knockdown C1498 cells were seeded in the upper chambers in 1 mL of medium per insert. Images of the scratched areas in the lower chambers were captured at 0 and 24 h. Wound areas were measured using ImageJ, and the wound closure rate was calculated using the following formula: wound closure rate (%) = (area at 0 h – area at 24 h) / area at 0 h × 100%.

### RNA extraction and qRT-PCR

Total RNA was extracted from C1498 leukemia cells using TRIzol Reagent (Thermo Fisher, USA) according to the manufacturer's instructions. Briefly, after cell lysis and chloroform addition, the upper aqueous phase was collected. RNA was precipitated with isopropanol, washed with ethanol, and finally dissolved in RNase-free water. The purified RNA was then reverse-transcribed to cDNA, which served as a template for qRT-PCR using Universal SYBR qPCR Mix. GAPDH was used as the endogenous control. The primer sequences are listed as follows: ITGB3-F GTGAGTGCGATGACTTCTCCTG; ITGB3-R CAGGTGTCAGTGCGTGTAGTAC; PCDH10-F GCTCTAAGGACAGTGGTCATGG; PCDH10-R CCAGTGCTTTACATTCCTCGGTG; VIM-F GCCAGCAGTATGAAAGCGTG; VIM-R: ACCTGTCTCCGGTACTCGTT.

### Statistical analysis

All experiments were performed independently at least three times. Data are presented as mean ± standard deviation (SD). Differences between two groups were assessed using unpaired *t*-test, while comparisons among multiple groups were conducted via one-way analysis of variance (ANOVA). *p* values were denoted as follows: ^*^ indicated *p* < 0.05; ^**^ indicated *p* < 0.01; ^***^ indicated *p* < 0.001; ^****^ indicated *p* < 0.0001. A *p* value < 0.05 was considered statistically significant.

## Results

### AML infiltration and chemotherapy synergistically impair testicular architecture and spermatogonia function

**Figure 1:**
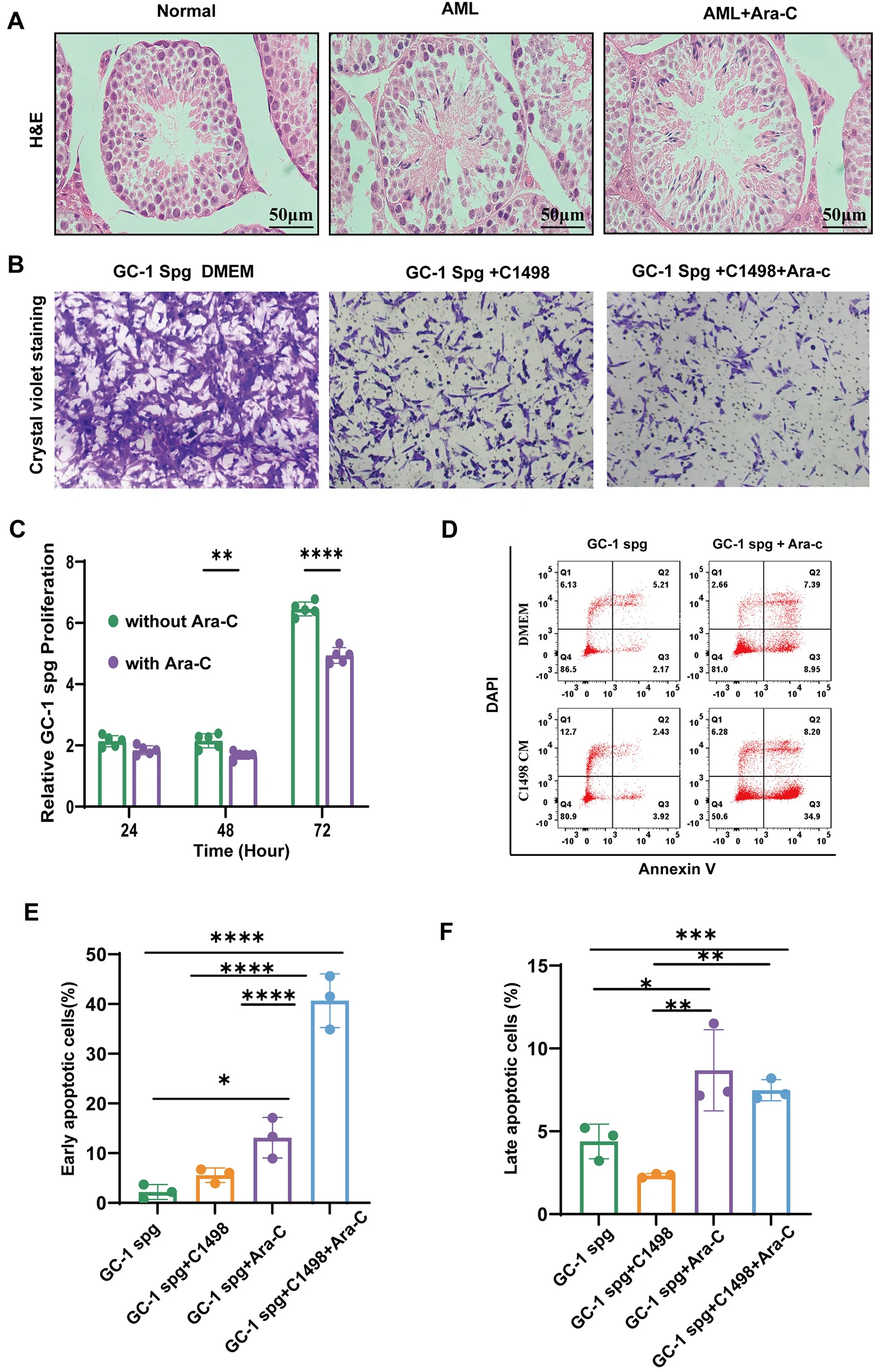
AML infiltration and chemotherapy synergistically impair testicular architecture and spermatogonia function. **(A)** Representative H&E staining images of testicular sections from the normal mice, AML-infiltrated mice, and AML-infiltrated mice treated with Ara-C. Scale bar = 50 um. **(B)** Representative images of GC-1 spg cell migration following treatment with DMEM, C1498-conditioned medium (CM), or C1498-CM plus Ara-C, as detected by crystal violet staining. **(C)** Growth curves of GC-1 spg cells treated with or without Ara-C treatment. **(D)** Representative flow cytometry plots of GC-1 spg cell apoptosis. Cells were treated with DMEM, Ara-C, C1498-CM or C1498-CM plus Ara-C. **(E, F)** Quantification of **(E)** early apoptotic and **(F)** late apoptotic GC-1 spg cell populations following exposure to DMEM, Ara-C, C1498-CM or C1498-CM plus Ara-C. Significance was assessed by unpaired *t*-test **(C, E, F)**. *p < 0.05, **p < 0.01, ****p < 0.0001 vs. control.

To investigate the impact of leukemia infiltration and chemotherapy on testicular integrity, we established an acute myeloid leukemia (AML) mouse model by intravenously injecting 1^*^10^6^ C1498 AML cells via the tail vein. Histopathological examination revealed that both the AML-alone and AML+Ara-C groups exhibited marked reductions in spermatogenic cell numbers and disrupted seminiferous tubule architecture compared to controls (Figure 1A). Notably, the co-exposure group (AML+Ara-C) showed more pronounced impairments (Figure 1A), suggesting that chemotherapeutic intervention may exacerbate testicular dysfunction under AML infiltration.

To further elucidate the impact of the AML microenvironment and chemotherapy on spermatogonial migration, we utilized a transwell co-culture system to mimic leukemic infiltration *in vitro*. Co-culture with C1498 AML cells significantly reduced the migration of GC-1 spg spermatogonial cells, an effect that was further exacerbated by Ara-C administration (Figure 1B). Simultaneously, Ara-C treatment suppressed proliferation and induced apoptosis in GC-1 spg cells; notably, these anti-proliferative and pro-apoptotic effects were substantially enhanced in the presence of C1498 cells (Figures 1C–F). Collectively, these findings suggest that leukemia infiltration and chemotherapeutic agents not only independently impair germ cell function but also synergistically amplify testicular damage.

### SPAG6 characterization as a cancer-testis antigen with dysregulated expression during AML progression

**Figure 2:**
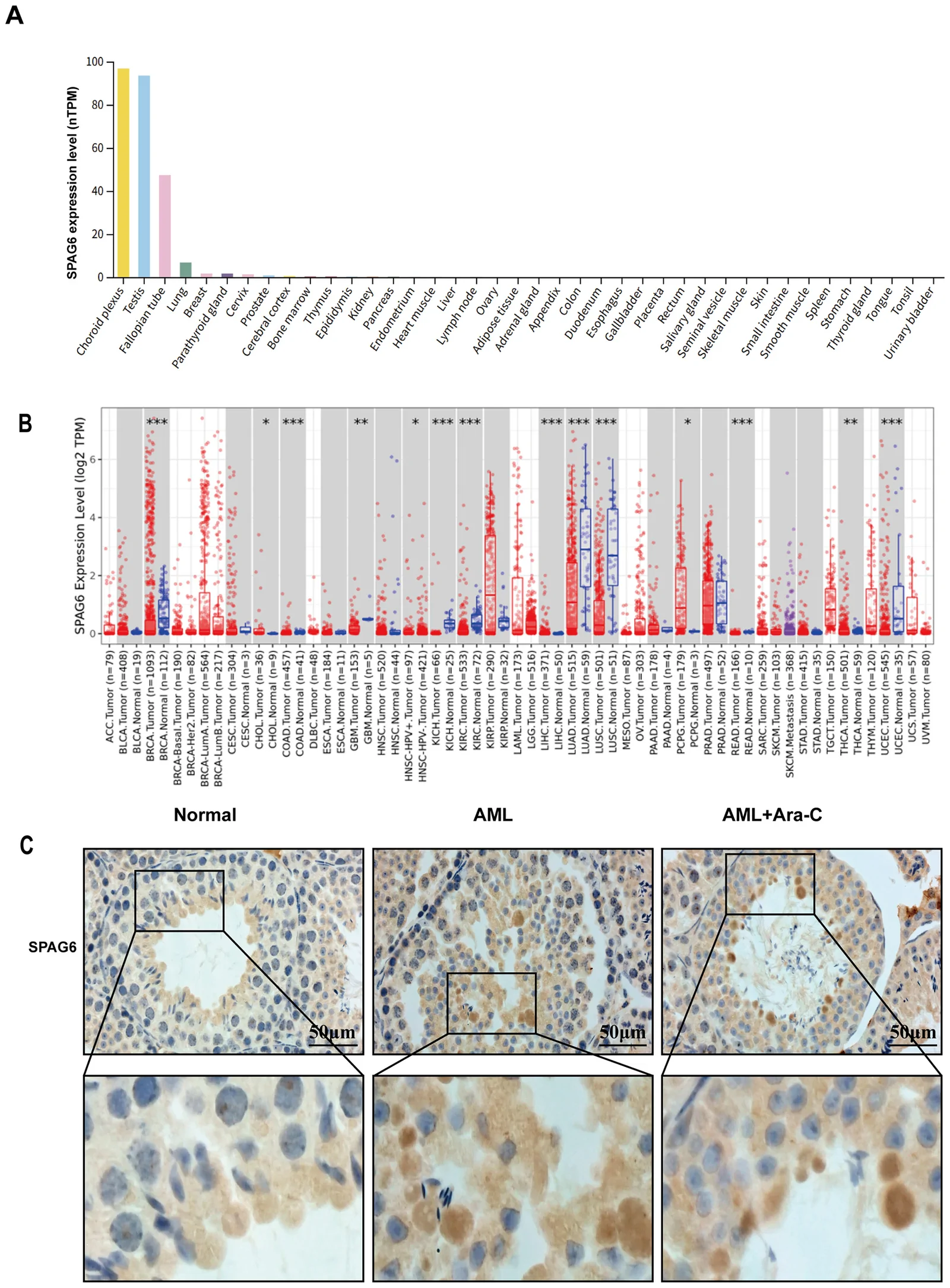
SPAG6 characterization as a cancer-testis antigen with dysregulated expression during AML progression. **(A)** SPAG6 expression profiles in normal human tissues from the Human Protein Atlas database. **(B)** Pan-cancer expression profile of SPAG6. Box plots represent *SPAG6* mRNA expression levels (log_2_ TPM) across various tumor types (red) and corresponding adjacent normal tissues (blue) obtained from the TIMER database. **(C)** Representative immunohistochemical (IHC) staining of SPAG6 in testicular tissue from normal mice, AML-infiltrated mice, and AML-infiltrated mice treated with Ara-C. Scale bar = 50 um. Significance was assessed by unpaired *t*-test **(B)**. * *p* < 0.05, ** *p* < 0.01, *** *p* < 0.001 vs. control.

We then assess the expression pattern of SPAG6. Consistent with previous study, Figure 2A shows that SPAG6 was predominantly expressed in reproductive tissues, including testis, while pan-cancer analysis showed that SPAG6 expression correlates with prognosis in various malignancies, including AML (Figure 2B).

This dual enrichment—upregulation in AML cells and high physiological abundance in the testes—led us to hypothesize that SPAG6 may mediate the preferential chemotaxis and colonization of AML cells within the testicular niche. To test this hypothesis, we examined SPAG6 expression in our established AML mouse model. Immunohistochemical analysis showed that while SPAG6 was constitutively expressed in normal testicular tissues, its levels were further significantly elevated following leukemic infiltration and post-chemotherapy (Figure 2C). These findings suggest that SPAG6 expression in the testicular microenvironment is dynamically upregulated concomitant with leukemic infiltration and chemotherapeutic intervention.

### SPAG6 deficiency attenuates leukemic proliferation and multi-organ infiltration *in vivo*

**Figure 3:**
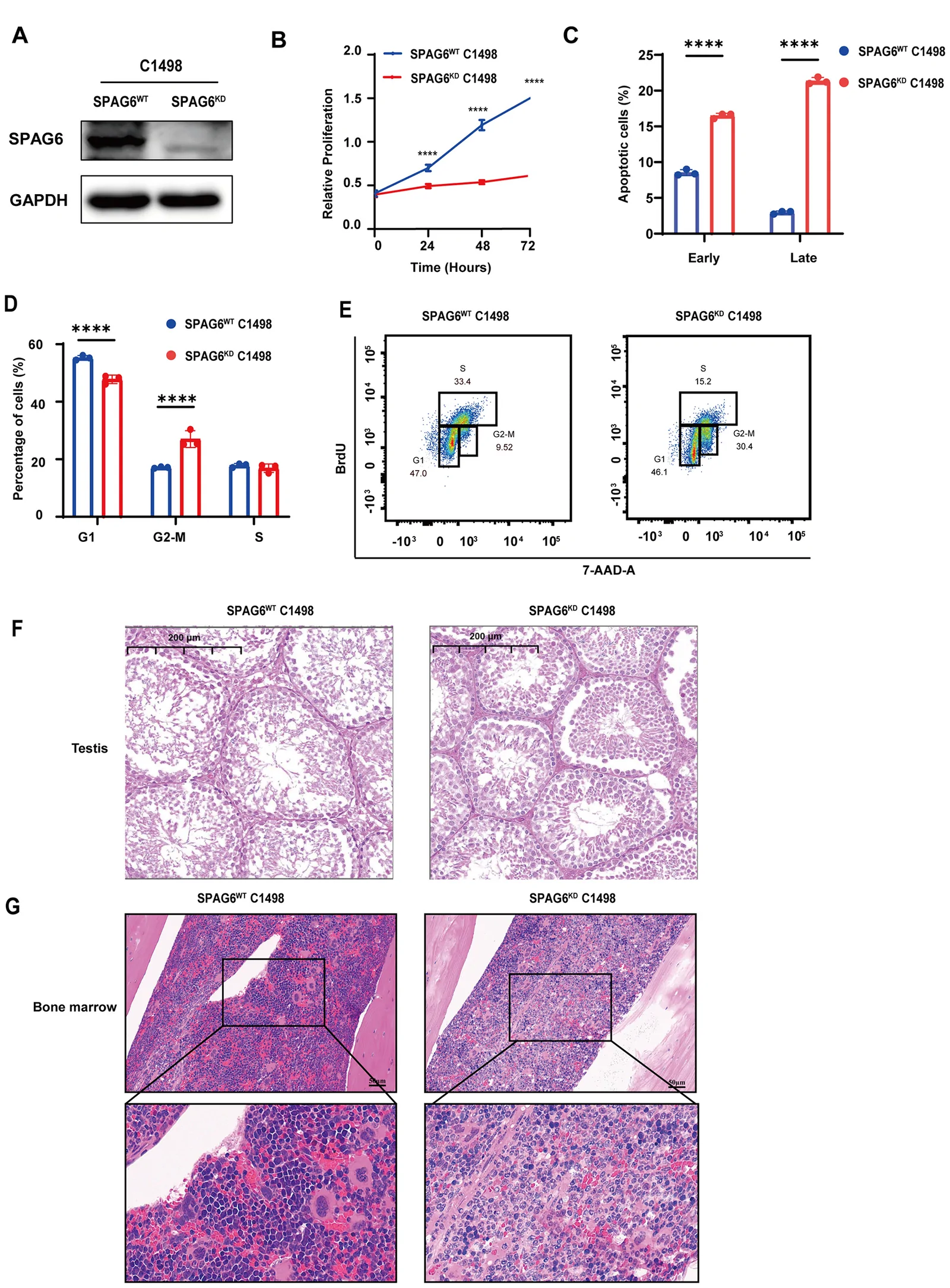
SPAG6 deficiency attenuates leukemic proliferation and multi-organ infiltration *in vivo*. **(A)** Western blot analysis of SPAG6 protein levels in control and SPAG6-knockdown (KD) C1498 cells. **(B)** Proliferation rate of control and SPAG6-KD C1498 cells measured by CCK-8 assay. **(C)** Quantification of early and late apoptotic cells in control and SPAG6-KD C1498 cells. **(D, E)** Cell cycle distribution in control and SPAG6-KD C1498 cells. **(D)** Quantification of cell cycle phases and **(E)** representative flow cytometric histograms. **(F, G)** Assessment of leukemic infiltration *in vivo*. Representative H&E staining of testicular sections (Scale bar = 200 um) **(F)** and bone marrow (Scale bar = 50 um) **(G)** from mice intravenously injected with control or SPAG6-KD C1498 cells. Significance was assessed by unpaired *t*-test **(B, C, D)**. ****p < 0.0001 vs. control.

To further investigate the functional role of SPAG6 in leukemia progression, we established stable SPAG6-knockdown C1498 cell lines via shRNA-mediated silencing (Figure 3A). SPAG6 deficiency was associated with reduced cell proliferation and concurrently increased apoptosis in C1498 cells (Figures 3B, C). Consistently, cell cycle analysis via flow cytometry revealed that SPAG6 knockdown resulted in a significant accumulation of cells in the S-phase, accompanied by a concomitant reduction in the G1 and G2/M populations (Figures 3D, E). To further assess the impact of SPAG6 on leukemic infiltration *in vivo*, equal numbers of wild-type (WT) or SPAG6-knockdown (KD) C1498 cells were intravenously injected into C57BL/6J mice. Histopathological analysis showed that SPAG6-KD recipients exhibited significantly reduced leukemic infiltration in both testicular tissue and bone marrow compared to WT recipients (Figures 3F, G). These results demonstrate that SPAG6 is essential for leukemic cell proliferation and tissue infiltration.

### Transcriptomic analysis reveals SPAG6 regulates adhesion and migration pathways in AML

**Figure 4:**
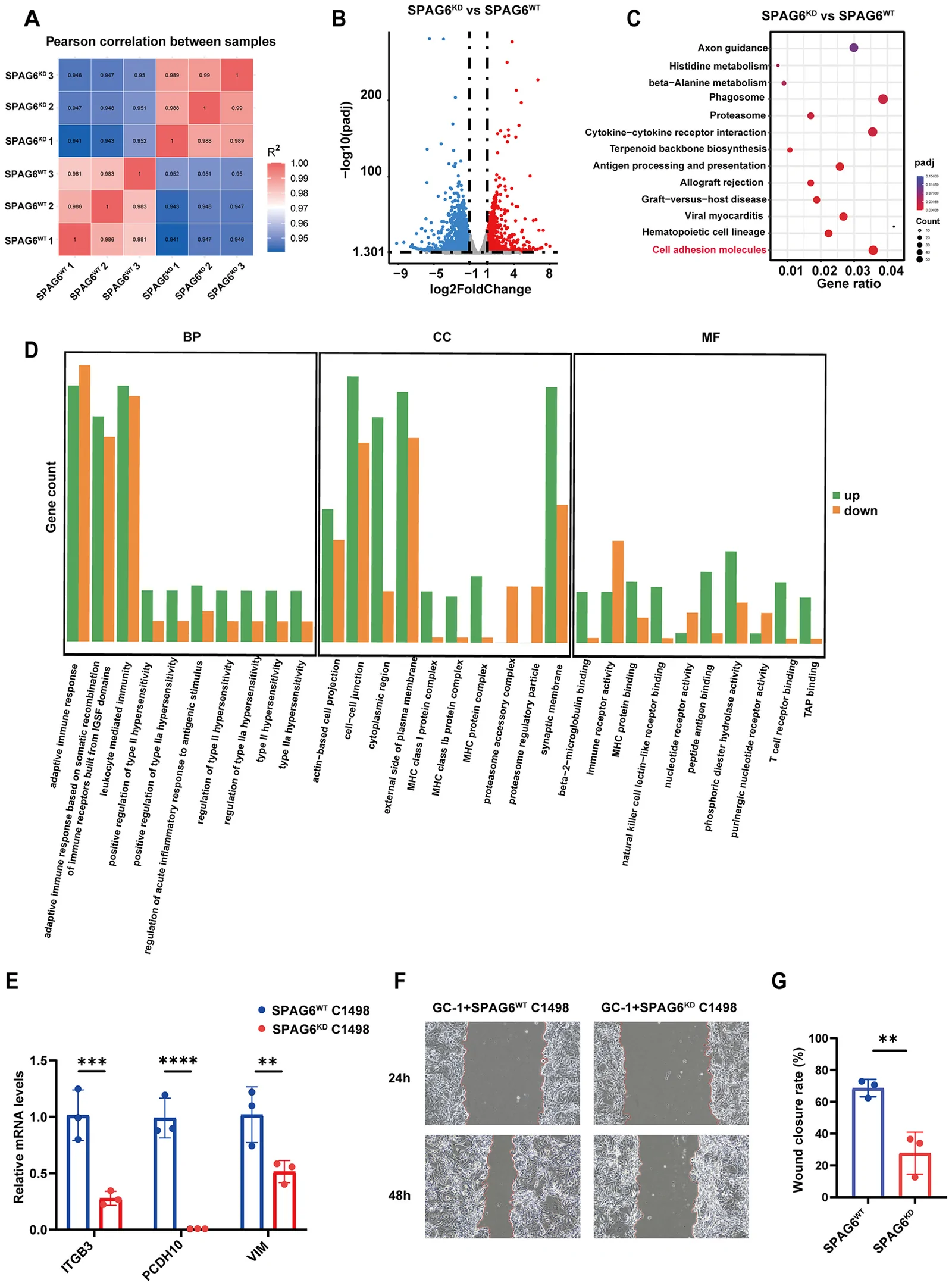
Transcriptomic analysis reveals SPAG6 regulates adhesion and migration pathways in AML. **(A)** Pearson correlation heatmap of RNA-seq data from SPAG^WT^ and SPAG6^KD^ C1498 cells. **(B)** Volcano plot of differentially expressed genes (DEGs) between SPAG^WT^ and SPAG6^KD^ C1498 cells. **(C)** KEGG pathway enrichment analysis of DEGs. **(D)** Gene Ontology (GO) enrichment analysis of DEGs categorized by Biological Process (BP), Cellular Component (CC), and Molecular Function (MF). **(E)** Relative mRNA levels of ITGB3, PCDH10, and VIM in SPAG^WT^ and SPAG6^KD^ C1498 cells. **(F)** Representative microscopic images of the scratch wound healing assay for GC-1 spg cells after 24 h of co-culture with SPAG^WT^ and SPAG6^KD^ C1498 cells. **(G)** Quantification of wound closure rates in the SPAG^WT^ and SPAG6^KD^ co-cultured groups. Significance was assessed by unpaired t-test **(E, G)**. **p < 0.01,***p < 0.001, ****p < 0.0001 vs. control.

To elucidate the molecular mechanisms underlying SPAG6 function in AML, we performed RNA sequencing (RNA-seq) on SPAG6-KD and SPAG6-WT C1498 cells. Pearson correlation analysis revealed good reliability of the sequencing data and differential expression analysis identified 1,093 upregulated and 1,963 downregulated genes in SPAG6-KD cells compared to WT controls (Figures 4A, B). KEGG pathway enrichment analysis revealed that differentially expressed genes (DEGs) were significantly enriched in cell adhesion molecule pathways (Figure 4C). Gene Ontology (GO) enrichment analysis further categorized DEGs into Biological Process (BP), Cellular Component (CC), and Molecular Function (MF) terms. Within the Biological Process, up-regulated genes in SPAG6-KD cells were enriched in immune-related pathways, including adaptive immune response, leukocyte-mediated immunity, and regulation of type II hypersensitivity (Figure 4D). Cellular Components revealed that SPAG6 depletion affected terms related to the cell structure and interaction, such as actin-based cell projection, cell-cell junction, and cytoplasmic region, suggesting impaired cellular adhesion machinery upon SPAG6 depletion. Taken together, these data indicate that SPAG6 may regulate leukemic cell migration primarily through modulation of cell adhesion pathways. Consistent with the RNA-seq analysis, qRT-PCR further confirmed that knockdown of SPAG6 significantly reduced the expression of cell adhesion-related genes, including ITGB3, PCDH10, and VIM (Figure 4E). To functionally validate these transcriptomic findings, we performed a transwell-based scratch wound healing assay. The migratory capacity of GC-1 spg cells was reduced when co-cultured with SPAG6-KD C1498 cells compared to controls (Figures 4F, G). Besides, our previous study demonstrated that SPAG6 overexpression significantly enhanced the migratory capacity (approximately 1.8% *vs*. 1.0% in controls) of leukemia cells (15). These functional results corroborate the RNA-seq findings, supporting the role of SPAG6 in regulating cell adhesion and migration pathways.

### SPAG6 deficiency exacerbates chemotherapy-induced oxidative stress and mitochondrial dysfunction in spermatogonial cells

**Figure 5:**
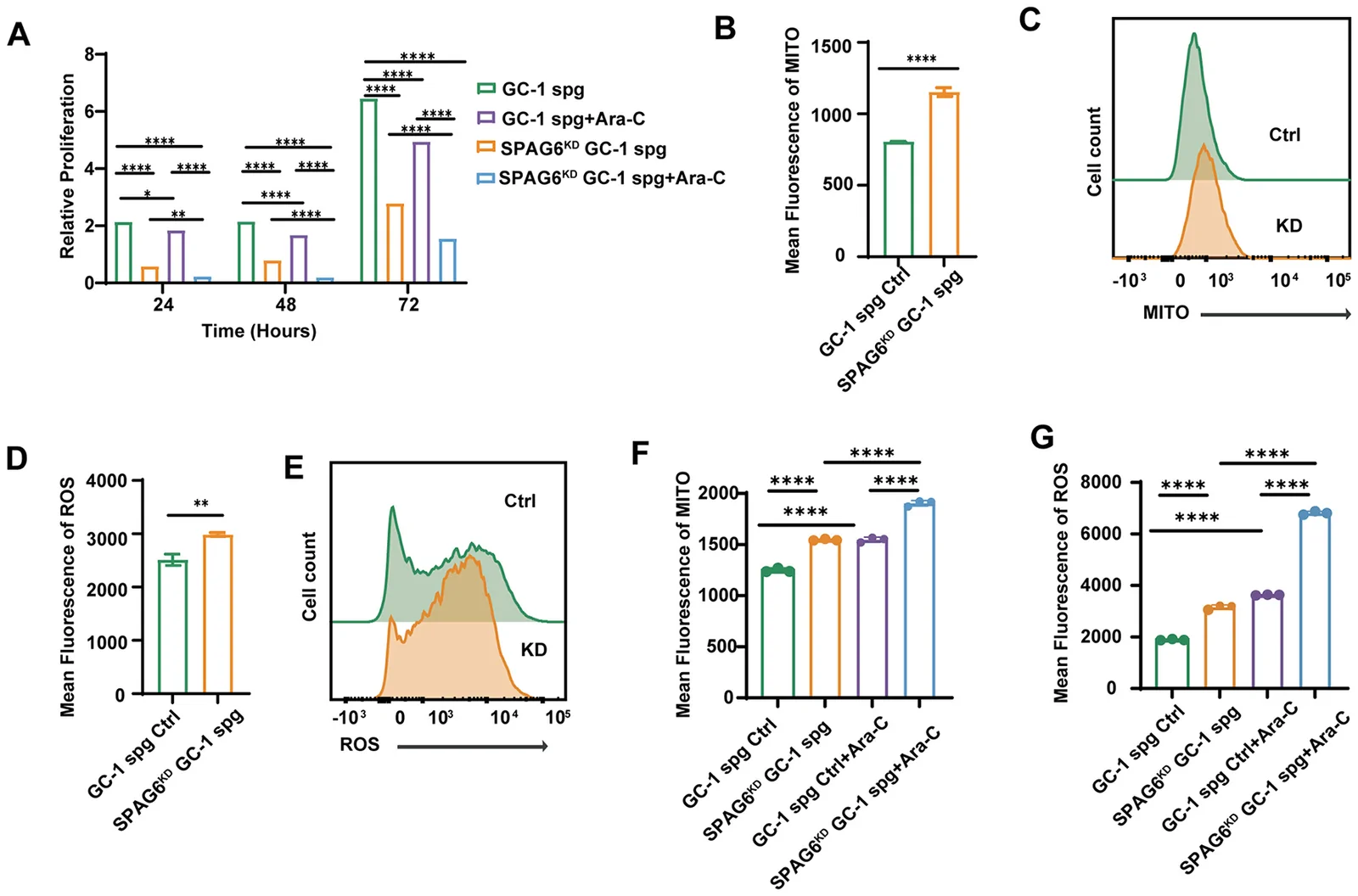
SPAG6 deficiency exacerbates chemotherapy-induced oxidative stress and mitochondrial dysfunction in spermatogonial cells. **(A)** Proliferation rate of WT and SPAG6-KD GC-1 spg cells treated with or without Ara-C at 24, 48, and 72 h measured by CCK-8 assay. **(B, C)** Assessment of mitochondrial activity membrane potential via MitoTracker staining. **(B)** Quantification of mean fluorescence intensity (MFI) and **(C)** Representative flow cytometry histogram between WT and SPAG6-KD GC-1 spg cells with or without Ara-C treatment. **(D, E)** Evaluation of intracellular ROS levels. **(D)** Quantitative analysis of ROS MFI and **(E)** representative flow cytometric histograms between WT and SPAG6-KD GC-1 spg cells with or without Ara-C treatment. Significance was assessed by unpaired *t*-test **(A, B, D, F, G)**. * *p* < 0.05, ** *p* < 0.01, **** *p* < 0.0001 vs. control.

Given that SPAG6 is predominantly expressed in the testes and is upregulated during AML infiltration and chemotherapy, we next evaluated the functional role of SPAG6 in GC-1 spg spermatogonia under chemotherapeutic stress. SPAG6 knockdown significantly suppressed GC-1 spg cell proliferation, an effect further enhanced by Ara-C treatment (Figure 5A). Given that testicular cells are rich in mitochondria and highly sensitive to oxidative injury (5, 18), we assessed mitochondrial membrane potential and intracellular ROS production to further characterize the cellular stress response associated with SPAG6 depletion and Ara-C treatment. Under basal conditions, SPAG6-KD spermatogonial cells exhibited significantly elevated mitochondrial membrane potential and ROS levels compared to control cells (Figures 5B–E). Upon Ara-C treatment, these effects were markedly exacerbated, with SPAG6-KD cells showing higher mitochondrial membrane potential and ROS levels than control cells (Figures 5F, G). These observations indicate that SPAG6 depletion is associated with redox imbalance and heightened oxidative stress in germ cells, in contrast to its pro-tumorigenic role in leukemia cells.

## Discussion

Currently, the infiltration of hematological malignancies into the reproductive system and the compounded effects of chemotherapy on fertility remain poorly defined (19, 20). Although recent advances have improved AML survival, the physiological impact of leukemia and chemotherapy on male germ cells has received limited attention (4). Building on our previous identification of SPAG6 as a prognostic biomarker and a critical driver of AML progression (15, 17, 18), the current study reveals SPAG6 exerts dual role within testicular microenvironment, promoting pro-invasive leukemia infiltration and protecting against chemotherapy-induced testicular injury. According to Paget's “Seed and Soil” theory, the site-specific infiltration of leukemia cells depends on the crosstalk between tumor cells (the seed) and the organ microenvironment (the soil) (21, 22). In our study, the concurrent high expression of SPAG6 in both AML cells and testicular tissue suggests a molecular affinity that facilitates the homing and colonization of leukemic cells to the testes." Transcriptomic profiling further underscored that SPAG6 facilitates this organ-specific infiltration by modulating cell adhesion and junctional signaling pathways.

Our findings reveal that SPAG6 may exert cell-type-dependent, opposing functions in the testis during AML: it is associated with increased leukemia cell proliferation and testicular infiltration while simultaneously reducing chemotherapy-induced oxidative damage to spermatogonia. Mechanistically, our transcriptomic analysis in C1498 leukemia cells indicates that SPAG6 may maintain cellular structural integrity and mediate cell-cell interactions, thereby facilitating adhesion and migration toward the testis. In contrast, in spermatogonia, SPAG6 appears to operate within a critical antioxidant network, where its loss triggers ROS accumulation, mitochondrial dysfunction, and proliferative arrest. we propose that this “functional switch” is rooted in the intrinsic biological differences between malignant leukemia cells and normal germ cells. These differences likely encompass variations in baseline mitochondrial content, differential susceptibilities to oxidative stress, distinct proliferative dynamics, and the presence of cell-type-specific SPAG6-interacting partners. While the precise molecular mediators warrant further investigation, this framework underscores a crucial clinical implication: future therapeutic strategies must carefully balance the desired anti-leukemic efficacy with potential cytotoxic impacts on the reproductive system.

Various chemotherapeutic agents impair mitochondrial electron transport and disrupt the mitochondrial membrane potential (MMP), leading to ROS overproduction, which in turn impairs mitochondrial function and exacerbates oxidative stress (23, 24). Testicular cells, being highly dependent on mitochondrial function and rich in mitochondria, are particularly susceptible to chemotherapy-induced ROS elevation and mitochondrial dysfunction (25). In our study using GC-1 spg spermatogonial cells, we found that SPAG6 knockdown concurrently increased both ROS levels and MMP; importantly, this vulnerability was significantly amplified under chemotherapeutic stress. Consistent with our findings, Luo et al. reported that SPAG6 knockdown in leukemia cells also elevated ROS and MDA levels, whereas SPAG6 overexpression or treatment with the antioxidant N-acetylcysteine attenuated these effects (18). These findings collectively indicate that SPAG6 is essential for maintaining mitochondrial homeostasis and antioxidant capacity. However, the precise mechanism by which SPAG6 deficiency leads to increased ROS production and mitochondrial dysfunction remain incompletely understood. Given the well-established role of SPAG6 in maintaining cytoskeletal integrity and microtubule stability (10), we propose a potential mechanistic link: microtubules serve as essential tracks for mitochondrial trafficking. SPAG6 deficiency may impair mitochondrial transport, dynamics, or proper subcellular distribution. Such spatial and functional disruptions could subsequently trigger excessive ROS generation and initiate a vicious cycle of mitochondrial dysfunction. Additionally, SPAG6 may directly or indirectly interact with mitochondrial antioxidant proteins or regulate their expression, providing another layer of ROS control. The exact molecular mechanisms driving this SPAG6-ROS-mitochondria axis warrant comprehensive investigation in future studies.

There are several limitations in our study that warrant further investigation. First, as our results are based solely on murine models and cell lines, subsequent translational studies utilizing human clinical cohorts are imperative to validate the role of SPAG6 in leukemic testicular infiltration and chemotherapy-induced fertility impairment in patients. Second, our results demonstrate that AML infiltration combined with Ara-C chemotherapy exacerbates testicular damage and induces a more pronounced upregulation of SPAG6 expression. However, since we did not include an Ara-C-alone group, the relative contributions of leukemic infiltration vs. chemotherapy to testicular damage and SPAG6 elevation remain unclear. Future studies with an Ara-C-alone group are needed to dissect these individual and interactive effects. Besides, our experimental design relied primarily on loss-of-function models. Future investigations incorporating overexpression or genetic rescue experiments are essential to definitively confirm that the observed cellular defects are specific consequences of SPAG6 depletion rather than potential off-target effects. Finally, our assessment of the BTB was limited to H&E-based morphological observations and basic proliferation analyses. To gain a more comprehensive understanding, future studies should employ specific functional assays to evaluate BTB integrity and permeability (e.g., using tracer assays), examine the expression of tight junction proteins, and dynamically monitor overall spermatogenic function.

In conclusion, this study demonstrates that SPAG6 may exert cell-type-specific, opposing functions in AML-infiltrated testes: it is associated with leukemic cell infiltration while potentially protecting spermatogonial cells from chemotherapy-induced oxidative injury. As AML survival outcomes continue to improve, the preservation of reproductive health must be prioritized alongside anti-tumor efficacy. Future research should focus on developing targeted therapies that selectively antagonize SPAG6 in malignant cells while co-administering localized antioxidants to safeguard the spermatogonia from oxidative stress.

## Data Availability

The data presented in the study are deposited in the NCBI SRA repository, accession number PRJNA1513745.
